# An intersectional approach to exploring lived realities and harnessing the creativity of ethnic minority youth for health gains: protocol for a multiphase mixed method study

**DOI:** 10.1186/s12889-023-16011-0

**Published:** 2023-06-09

**Authors:** Rodrigo Ramalho, Vartika Sharma, Renee Liang, Rachel Simon-Kumar, Shanthi Ameratunga, Arier Lee, Kristy Kang, Roshini Peiris-John

**Affiliations:** 1 Section of Social and Community Health, School of Population Health, The University of Auckland, Auckland, New Zealand; 2 Section of Epidemiology and Biostatistics, School of Population Health, The University of Auckland, 28 Park Avenue, Grafton, Auckland, 1023 New Zealand

**Keywords:** Young people, Wellbeing, Intersectionality, Co-design, Participatory research, Minority, Ethnic minority, Creative arts, Aotearoa, New Zealand

## Abstract

**Background:**

Understanding the diversity and multiplicity of identities experienced by youth in Aotearoa (Te reo Māori name of the country) New Zealand (NZ) is vital to promoting their wellbeing. Ethnic minority youth (EMY) in NZ (defined as those identifying with Asian, Middle Eastern, Latin American and African ethnic origins) have been historically under-studied and under-counted, despite reporting high levels of discrimination, a major determinant of mental health and wellbeing and potentially a proxy for other inequities. In this paper, we describe the protocol for a multi-year study that examines, using an intersectional approach, how multiple marginalised identities impact mental and emotional wellbeing of EMY.

**Methods:**

This is a multiphase, multi-method study designed to capture the diversity of lived realities of EMY who self-identify with one or more additional marginalised intersecting identity (the population referred here as EMYi). Phase 1 (Descriptive study) will involve secondary analyses of national surveys to examine the prevalence and relationships between discrimination and wellbeing of EMYi. Phase 2 (Study on public discourse) will analyse data from media narratives, complemented by interviews with stakeholders to explore discourses around EMYi. Phase 3 (Study on lived experience) will examine lived experiences of EMYi to discuss challenges and sources of resilience, and how these are influenced by public discourse. Phase 4 (Co-design phase) will use a creative approach that is youth-centered and participatory, and will involve EMYi, creative mentors and health service, policy and community stakeholders as research partners and advisors. It will employ participatory generative creative methods to explore strengths-based solutions to discriminatory experiences.

**Discussion:**

This study will explore the implications of public discourse, racism and multiple forms of marginalisation on the wellbeing of EMYi. It is expected to provide evidence on the impacts of marginalisation on their mental and emotional wellbeing and inform responsive health practice and policy. Using established research tools and innovative creative means, it will enable EMYi to propose their own strength-based solutions. Further, population-based empirical research on intersectionality and health is still nascent, and even more scarce in relation to youth. This study will present the possibility of expanding its applicability in public health research focused on under-served communities.

## Backround

Ethnic minority groups are a historically under-studied population in Aotearoa New Zealand (NZ) [1, 2] and internationally [3, 4]. In 2018, 20% of NZ’s youth population comprised those identifying with Asian, Middle Eastern, Latin American and African ethnic origins (referred to in this paper as ‘ethnic minorities’) [5]. Ethnic minorities in NZ reside as citizens, permanent residents, international students, recent migrants and/or refugees. Existing research points to high rates of discrimination, bullying and psychological distress among ethnic people, leading to an increased impetus for policy-relevant research [6–10]. Health and wellbeing experiences typically have been discussed as an outcome of ethnicity, as if being an ‘Asian’ or ‘Middle Eastern’ has an isolated effect. However, a singular social dimension, viz., ethnicity, seldom has a segregated impact on populations; rather, health and wellbeing experiences are an outcome of an intersection of social identities.

**Fig. 1:**
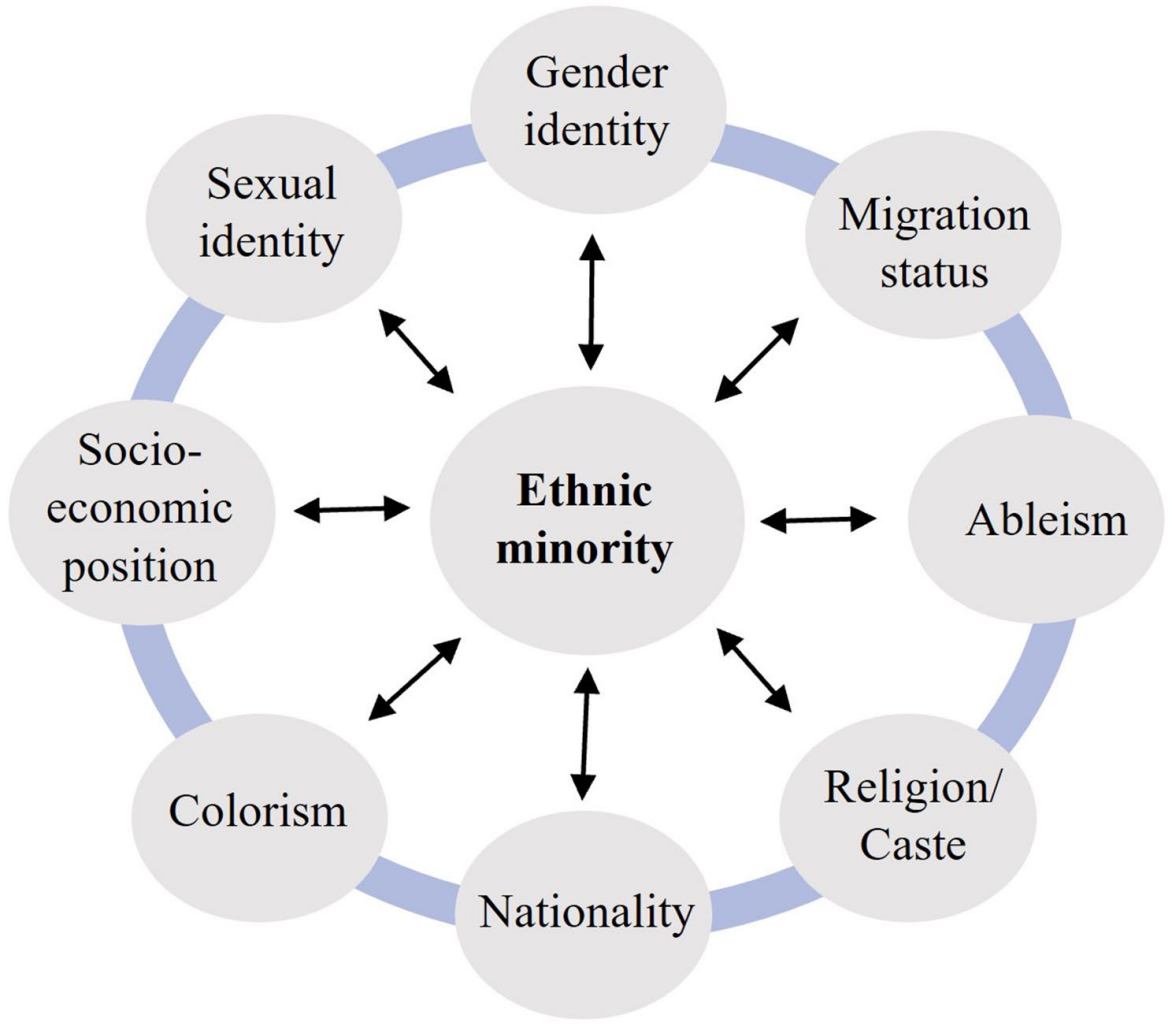
Intersecting axes of minority status

Young people can identify with multiple social markers such as race and ethnicity, diverse gender identities and sexualities, abilities and disabilities, and socio-economic status, among other social axes (Fig. 1). These identities interact with each other and the broader socio-cultural and political environments they live in acting, effectively, as upstream determinants often also manifesting as multiple levels of oppression [11].

Therefore, it is not surprising that intersectionality, originating in black feminist scholarship [12], is increasingly seen as a promising approach to analyse the processes and power structures that result in health inequities. By encompassing the vast heterogeneity in people’s lived experiences, this approach attends to multiple disadvantages that define several under-served population groups [13]. Thus, it triggers the development of potentially transformative interventions to address ongoing and emerging inequities [13, 14].

Despite advances in intersectionality theory, population-based empirical research on intersectionality and health is still nascent, and even more scarce in relation to youth. Where it exists, research shows intersectional populations as groups especially susceptible to multiple social obstacles faring much worse than single-axis disparities indicate [15–17]. For example, Chinese students in NZ who also identified as sexual/gender minorities had even poorer mental health outcomes compared to sexual/gender majority [17]. In 2019, a quarter of ethnic minority adolescents in NZ reported depressive symptoms [18]. Similarly, Māori and Pasifika youth in NZ also report more health risks if they identify with other minority population groups such as gender/sexual minorities or youth with disabling conditions [19].

Against this backdrop, the overarching aim of the present study is to examine EMY’s lived experiences in the intersections of multiple identities, their experiences of inclusion/exclusion and the role of public discourse, the implications for their wellbeing, and possibilities for transformations of EMY lived experiences so as to flourish in their multiple identities. Taking an intersectionality perspective, this study addresses these complex layers of identity formation, marginalisation, and wellbeing through a multi-method, multi-epistemic research design that encompasses conventional quantitative-qualitative tools and innovative creative techniques.

The study outcomes will contribute towards growing the scholarship around EMY mental health. First, this study will help improve understanding of intersectional forms of stigma and discrimination (of racism, sexism, able-ism, heteronormativity, etc.) for already marginalised minority youth (EMYi) and the distinctive ways these coalesce to contribute to unique set of health inequities among minority youth. Second, the study will provide the much-required evidence about how youth-led creative art forms can contribute towards shaping health policy and practice. The study will thus provide research-based evidence to facilitate improvements in the delivery of health care and community services, integrating concerns of ethnic and other issue-based communities (e.g., rainbow communities or disability communities). Leading from this, the study will offer insights into systems of discrimination – both mainstream and from their own communities – and strategies to develop healthcare interventions that are not solely culturally focused but also integrate other identity dimensions (or what we call ‘cultural + + competencies’). Third, we anticipate that this study will fill in the existing knowledge gap on the use of an intersectionality approach in public health research. Lastly, although the study is focused on the experiences of EMYi in NZ, the study findings will be relevant to other migrant-receiving countries which have a significant population of ethnic minority communities, who are likely to share similar lived experiences of marginalisation.

## Methods

Conceptually, the research methodology departs from conventional epidemiological frameworks that focus on aggregation and disaggregation of variables to understand public health outcomes. Instead, our study positions EMYi uniquely in the intersections of multiple marginalisations and also in relation to communities of relevance, whether it be a majority group (e.g. Pākehā (a Māori term for New Zealanders primarily of European descent) and Other European) or their own cultural groups. More specifically, the overall objectives of this study are to:

- Describe how EMYi flourish within, and are marginalised by their, multiple intersecting identities including in the ways they are represented in public discourse;
- Describe how multiple identities of marginalisation impact young people’s emotional wellbeing and mental health;
- Co-design creative works with self-identified EMYi to open conversations;
- Explore the applicability of intersectionality in public health research focused on some of the under-served communities in NZ.

### Study population

The population of interest in this study is EMY aged 16–24 years living in NZ who self-identify with one or more additional minority identity/ies. These include being from a sexual minority (bisexual, pansexual, gay or lesbian etc.); gender minority (transgender, gender diverse or cisgender woman); living with disability or a long-term health condition; from a poor socioeconomic position (based on individual, household- or neighbourhood-level indicators); being a migrant, or identifying with a religious minority. As our participants will self-identify their minority identity/ies, there is no limit to this list and participants are not, collectively, expected to be representative of any category of identity. We also acknowledge that minority identities are not fixed, and may vary depending on varying personal, social and political contexts.

As authors, we recognise our own positionalities as significant in developing this research study design and methods that will enable meaningful engagement with EMYi in NZ. The core research team, named co-authors on this paper, identify with diverse ethnic groups (Sri Lankan, Latin American, Chinese, Indian, and Korean) and with different migration histories (born in NZ/arrived as a migrant). Further, the research team has experience across several disciplines, including social science, public health, epidemiology, medicine and creative arts. Collectively, these attributes shaped the study design and methodology and will influence the data analysis and the way study outcomes are disseminated.

### Study design

**Fig. 2:**
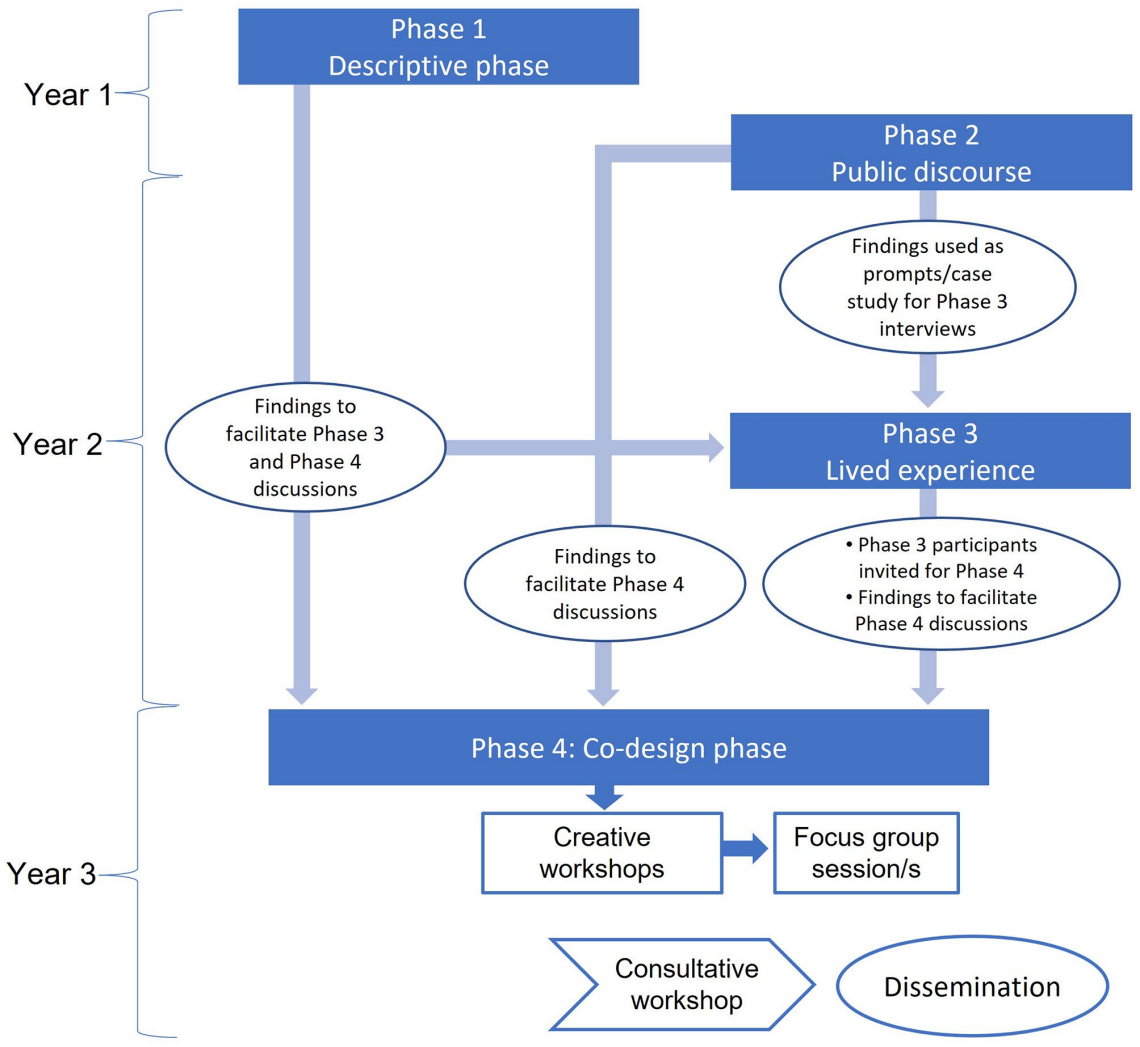
Overview of the study phases

This multi-method research, encompassing quantitative-qualitative tools and creative techniques, will be conducted over four phases across a three-year period (Fig. 2). All the study phases draw on key principles of community-based participatory research methods [20–22], and will provide us an opportunity to learn from EMYi who are knowledgeable about what affects their health and wellbeing and promote co-learning that focuses on social inequities; and subsequently integrate knowledge to facilitate changes for mutual benefit [23]. The four study phases are:

Phase 1: Descriptive quantitative study;

Phase 2: Study on public discourse;

Phase 3: Study of lived experience;

Phase 4: Co-design phase.

### Phase 1: descriptive quantitative study

This phase will provide a better understanding of the prevalence of experiences of discrimination, and of its impact on mental health and wellbeing among EMYi. We will conduct analysis of secondary data from multiple relevant national surveys as listed below:

- Youth2000 Surveys (2001, 2007, 2012, 2019): These surveys provide information on the health and wellbeing of NZ’s secondary school students (aged 13–19 years) [24–28].
- NZ Health Survey (NZHS) (2002/03, 2006/07, 2011/12, 2016/2017): These surveys provide information about the health and wellbeing of NZ’s non-institutionalised population aged 15 years and older, living in private dwellings [29].
- NZ General Social Survey (NZGSS) (2008, 2010, 2012, 2014, 2016, 2018): The NZGSS provides a profile of the wellbeing of people aged 15 years and over living in private dwellings in NZ [30].

These cross-sectional surveys with core items repeated across time allow us to examine experiences of discrimination in varying contexts (e.g., school, health care, police) alongside links between discrimination and health.

#### Key grouping variable

Each respondent will be allocated to ethnic groups with which they self-identify in their survey response. Participants who self-identify with any Asian, Middle Eastern, Latin American or African ethnicity (irrespective of any additional ethnicity) will be defined as EMY in this study. Young people who only identified as Pākehā (New Zealanders primarily of European descent) or Other European will be categorised as the ethnic majority reference group in quantitative analyses.

#### Exposure and outcomes

Experience of discrimination will be assessed through the self-reported experience of being treated unfairly because of ethnicity. Discrimination experienced by people identifying with some of the marginalised identities related to age, skin colour, race/ethnic group, gender, disability/health issue, sexual orientation, dress/appearance, accent/language spoken, and religious beliefs will be considered. Health measures of interest will include general wellbeing, emotional wellbeing, depressive symptoms, psychological distress and healthcare access and experience.

#### Data analyses

The patterns and distributions of self-reported experience of discrimination (including interactions with health, education and social sectors) and its relationship to measures of mental and emotional wellbeing of EMYi will be analysed for each survey. We will use descriptive statistics and relevant analytic approaches including logistic regression, ordinal logistic regression and mediation analysis taking into account survey design and sampling weights. Repeated surveys will allow us to explore changes over time.

### Phase 2: study on public discourse

In this phase, we will explore how EMYi are viewed, portrayed or referred to in public discourse, particularly in relation to the following dimensions: (i) identity (as New Zealanders) (ii) identity as ethnic minorities and as those with intersectional identities; and (iii) issues of health and wellbeing.

Data collection: Data will be collected using the following two key sources:

- *Media*: NZ-based print media will be used as the main source of data to examine representation of EMYi by others in the media. Using selected keywords, we will search and identify publications about or from EMYi over a pre-defined period. For consistency, only English language text will be used. Additionally, self-representation by EMYi will be explored on social media, for instance, on TikTok®.
- *Stakeholder interviews*: The media analysis will be complemented with information collected through semi-structured interviews with purposively selected key stakeholders, both from within and external to the ethnic communities. Potential stakeholders include key media persons and social media content creators identified during the media analysis, as well as youth leaders, subject matter experts, government officials, community leaders and Indigenous Māori (acknowledging lived realities of ethnic minorities in NZ as Te Tiriti o Waitangi/Treaty of Waitangi partners (Te Tiriti o Waitangi is a treaty with the English Crown that some Māori chiefs signed in 1840 that gave the Queen of England complete government over New Zealand; allowed Māori to maintain sovereignty over their lands, villages, properties, and treasures; and bestowed Māori with the same rights and privileges as British subjects)). We expect to achieve data sufficiency with a sample size of 12–15 interviews [31, 32].

#### Data analyses

Both the NZ-based print media and social media data will be analysed using an intersectional lens. To analyse NZ-based print media, we will use critical discourse analysis (CDA) to explore the public discourse on EMYi [33]. The analysis will follow three stages: (i) a description of the texts, (ii) interpretation of the process of production [who is saying it] and reception [to whom it is addressed], and (iii) explanation of the texts in light of wider sociocultural practices [34]. Interviews with key stakeholders will be analysed using thematic analysis [35]. The social media content will be analysed using media content analysis followed by in-depth interviews with young ethnic content creators about content creation and its consumption on social media.

### Phase 3: study of lived experience

In this phase, we will provide EMYi with a space to share and expand on their own lived experiences by exploring (i) processes of negotiating one’s identity/ies and flourishing; (ii) experiences of navigating social institutions, communities, and interpersonal dynamics; and (iii) the impact of marginalisation on health and mental and emotional wellbeing.

#### Recruitment and data collection

We will recruit a purposive sample of EMYi. Study recruitment will be promoted via various institutions’ email lists, including social clubs, schools, community groups, and places of religious worship; we will also use social media to distribute these advertisements and snowball sampling to reach under-served EMYi. We propose to recruit a sample of 15–20 participants; participants will no longer be recruited once data sufficiency is achieved [31, 32, 36]. At the time of recruitment, Phase 3 participants will be informed about the potential opportunity to participate in Phase 4. If interested, they will be contacted again once Phase 4 recruitment commences.

A semi-structured interview guide will be used to conduct one-on-one in-depth interviews for this phase. ‘Case studies’ based on the media analysis in Phase 2 might also be used to elicit insights and thoughts from the participants during the interview. All interviews will be conducted in English, and audio-recorded and transcribed verbatim for analysis. Whenever necessary, support will be provided to participants through research assistants competent in participants’ first language. Participants will be invited to edit their transcripts. This will include sufficient time to allow those for whom English is a second language to revise and expand the ways in which they wish to report their lived experiences.

#### Data analysis

Using an intersectional lens and a descriptive phenomenological approach, data will be analysed at a latent level, i.e., beyond the manifest content of the text and focusing on underlying assumptions, conceptualisations and ideas. This will involve interpretative work to gain an in-depth understanding of participants’ everyday experiences of intersectional identities and what these experiences mean to them. At the same time, this interpretation will also acknowledge that the identities are multiple, layered and dynamic, and attuned to the distinct yet intersecting systems of oppression and resilience that shape their experiences. Once the themes are identified, named and defined, an analytic narrative will be developed, supported by the data.

### Phase 4: co-design phase

Using a participatory co-design process, this phase will provide EMYi a central role in the design and development of creative art forms. These art-forms are not expected to be an expression of individual lived experience of the participants but rather how they would like to represent their experience collectively as a group. These art forms could be of any genre - short videos (e.g. TikTok), plays, music, graffiti art, graphic designs, podcasts or any other art forms, dependent on resources and time constraints.

#### Recruitment

All Phase 3 participants who agreed to be contacted for Phase 4 will be invited to participate. It is envisaged that interviews from Phase 3, prompting self-reflection, will enhance the quality and quantity of the contributions to be made in Phase 4 [37]. If there is a significant drop-out (> 30%) at the time of initiation of Phase 4 activities, we will recruit new participants using the recruitment strategy used for Phase 3.

#### Data collection

Phase 4 will involve multiple points of engagement with participants to allow sharing, discussion, analysis, design and evaluation. These interactions will be organised in a culturally and emotionally safe space. In brief, this will involve participation in a series of design workshops where participants will be provided prompts/provocations to trigger potential ideas for a creative artwork which they will develop in groups. Findings from Phases 1, 2 and 3 will inform these prompts/provocations to be used during Phase 4. Each group will be provided with resources (hours, space and consumables) to explore, generate and edit their work. Creative mentors (led by RL and Borni Te Rangopai Tukiwaho, an Indigenous Māori creative practitioner with requisite skills in working with marginalised groups in the arts space in NZ) will provide resources, knowledge, theory and creative tools, whilst participants will drive the creative process as the ‘expert of their experience’ and be empowered to explore freely. Up to eight face-to-face workshops will be organised over two to four months for making art forms, interspersed with time for writing, reflecting, incubation, collecting ideas and materials, germination and development.

The research team will collaborate with the creative mentors to document the experiences and reflections of the participants as they engage in the co-design process. The participants will also collaborate with the research team in terms of keeping a record of the creative process and the formative assessment of the co-design process (described below). For example, they may choose to provide constant feedback, invite the research team to ‘showings’ at various waypoints, share their notes and working materials, or collect video or written diaries of responses/reflections as they discover things. To enhance the rigour of the process, and not hinder creativity, the documentation and assessment of the co-design sessions will be iterative and co-led by the participants.

On the last day of the workshops, we will invite all Phase 4 participants to a focus group session to share their overall experience as participants in the co-design phase of the project. The session, expected to be of 45–60 min duration, will be audio-recorded and transcribed verbatim.

#### Data analysis

This creative journey is expected to reveal a more complex, and layered exploration of EMYi experiences, identity-finding journeys, and mental health. The data collected in this phase, in the form of experiences and reflections of the co-design process, will be analysed thematically in an iterative process informed by a grounded theory approach [38]. Further, while there is no expectation of completed artwork, some of the results may be revealing and potentially useful to understand and share EMYi experience [29].

### Consultation workshop and symposium

To synthesise and reflect on the findings and outputs from Phases 1 to 4, a consultative workshop will involve participants, creative mentors, members from our youth advisory group, youth health researchers and Māori advisors. Those participants who consented to it will share their design outputs and discuss how they would like their work to be shared with wider stakeholder networks, such as policymakers, community-based organisations, youth health advocates or political leaders. This workshop will thus allow for a global discussion by facilitating a reflective conversation about lived experiences of EMYi and inviting everyone to be responsive to their needs and driving narratives.

A symposium will be organised towards the end of the project to discuss how study findings and the proposed solutions could facilitate more responsive services for EMYi in NZ. Other dissemination activities could include: a youth-facing website with infographic/videoed material; symposia, and/or webinar presentations to policymakers, health service providers and other community stakeholders; press releases; peer-reviewed publications; and conference presentations. The Phase 4 workshops and two-way communication with stakeholders will inform our decision on which of these are most effective. Outside of our study, Phase 4 participants can choose to take their creative projects further and will be given advice and support to do so.

#### Study status

Study status: A summary of all 4 phases of the study is presented in Table 1. The research activities were initiated in September 2021 and will continue till August 2024. Participant recruitment is currently in different stages for the three phases - Phase 2, 3 and 4.

**Table 1:** Summary of study methods across Phases 1–4

Study Phase	Data source	Analytical approach
Phase 1: Descriptive Phase	Secondary data analysis: Youth 2000 surveys, NZ Health Survey, NZ General Social Survey	Descriptive statistics and relevant analytic approaches including logistic regression, ordinal logistic regression and mediation analysis
Phase 2: Study on public discourse	NZ-based print media, social media	Critical discourse analysis, media content analysis
	Stakeholder interviews	Thematic analysis
Phase 3: Study on lived experience	In-depth interviews	Thematic analysis
Phase 4: Co-design phase	Co-design process	Grounded Theory approach

## Discussion

This is a youth-centered study, which includes a co-design approach to allow youth participation in interpreting research findings and translating them to outputs that will be relevant for themselves and key stakeholders. To ensure that young people, besides research participants, have the opportunity to feedback and inform the research processes, a youth advisory group (YAG) has been constituted for this project. The YAG members represent a diverse range of EMYi and will meet twice every year during the project duration. The research team will seek feedback from the YAG for recruitment, data collection procedures, data analysis, and dissemination of the art forms.

There is growing recognition of the need to encompass intersectionality in minority research but the methodologies for doing so are still in its early stages. The present study takes a multi-method, multi epistemic approach to studying experiences of marginalisation and flourishing among EMYi. Using an intersectionality approach allows for multiple perspectives of exclusions to be studied through mixed method and using multiple data sources: EMYi’s own perspectives, current public discourse, official statistics, etc. The creative element is a particular strength as it has the potential to facilitate cross-cultural understanding and shape the public discourse in NZ to overcome implications arising from racism and marginalisation.

However, we foresee certain challenges as we undertake different phases of this study. An overarching concern is regarding using the term ‘ethnic minority’ which has been used to represent a highly heterogenous group with multiple identities and memberships. As we undertake data analysis, we will be mindful of trying not to homogenise the experiences of this diverse group. We also acknowledge a potential variation in how intersectionality may be captured in each phase of the study and, thus, a potential lack of consistency through this methodology. Additionally, we anticipate that this study will generate much interest in participation from among EMYi who are in tertiary education settings, who live in Auckland, or have significant presence in social media networks. While this is a group of interest, we would undertake targeted recruitment to include the voices of under-served young people (such as those in non-professional work settings or in remote locations). However, we do acknowledge that it might be difficult to facilitate participation of some of these under-served groups (such as EMYi with moderate or severe forms of physical or mental disability or limited English language) considering the resource constraints of this study. While the secondary data sources identified for use in Phase 1 were deemed to be the best available for this study, data was not collected for this purpose and therefore will be limited in the way it can be used. There is also a risk of participant drop-out in Phase 4 which will involve activities across several months. To minimize this, the research team will invest time in building relationships with the participants and among the participants and setting the right expectations in terms of the processes involved and the expected outcome.

To conclude, using an intersectional approach will allow the research methods used in this study to account for several disadvantages that shape the lived experience, and subsequently the mental health and emotional wellbeing of EMYi in NZ. Further, participatory generative design research methods [39] will facilitate development of strengths-based solutions and acknowledge the power of minority communities to find their own solutions. The use of creative art forms will generate rich, multilayered, nuanced and compelling data to help inform existing health policies and programmes to engage these groups better. In turn, giving youth creative control can be expected to generate innovative responses inside and outside of the research space. These may contribute in unique ways to public discourse on marginalisation. Lastly, we envisage that this multi-phase, multi-methods study will help capture the diversity of lived realities, and progress scholarship on the use of intersectional approaches in public health research.

## Data Availability

Individual participant data collected for this study will not be made available as per the conditions of consent received from participants of the study, including that data will be accessed only by the research team.

## List of abbreviations

CDA: Critical Discourse Analysis
EMY: Ethnic Minority Youth
EMYi: Ethnic minority youth who self-identify with one or more additional marginalised intersecting identity
MELAA: Middle Eastern, Latin American and African
NZ: New Zealand
NZGSS: New Zealand General Social Survey
NZHS: New Zealand Health Survey
OECD: Organization for Economic Cooperation and Development
YAG: Youth Advisory Group
